# The impact of hyperinsulinemia on short-term prognosis in patients with hypertriglyceridemia-induced acute pancreatitis

**DOI:** 10.3389/fendo.2025.1646307

**Published:** 2025-09-19

**Authors:** Mengjun Wang, Lei Zheng, Long Qian, Maoqi Xu

**Affiliations:** ^1^ Traditional Chinese Medicine Professional Master Training Base, Anhui University of Traditional Chinese Medicine Wuhu Hospital, Wuhu, China; ^2^ The First Clinical Medical College, Anhui University of Traditional Chinese Medicine, Hefei, China; ^3^ General Surgery Department, Wuhu Hospital of Traditional Chinese Medicine, Wuhu, China

**Keywords:** acute pancreatitis, hypertriglyceridemia, hyperinsulinemia, short-term prognosis, endocrine

## Abstract

**Purpose:**

The short-term prognosis of hyperinsulinemia in patients with hyperlipidemia resulting in acute pancreatitis remains uncertain, so this research explores the correlation between them.

**Material and methods:**

This study retrospectively analyzed patients treated for hypertriglyceridemic acute pancreatitis at Wuhu Hospital of Traditional Chinese Medicine between January 2020 and April 2023. Patients were divided into two groups based on laboratory diagnosis: the hyperinsulinemia group (HINS) and the non-hyperinsulinemia group (NHINS). The study aims to evaluate the short-term effects of hyperinsulinemia on acute pancreatitis.

**Results:**

A total of 92 patients with Hypertriglyceridemic pancreatitis were included in the study before receiving lipid-lowering therapy, with 44 in the HINS group and 48 in the NHINS group. Significant differences were observed between the two groups in terms of white blood cell count (WBC), hypersensitive C-reactive protein (CRP) levels, and fasting blood glucose at 2, 3-4, and 5–7 days after initiation of lipid-lowering intervention (*P* < 0.05). Following admission, patients received symptomatic interventions. However, there was a significant difference in the rate of decrease compared to the baseline between the fifth and seventh days (*P* < 0.05). Additionally, the groups showed significant differences in the readmission rates for recurrent pancreatitis within 30 days post-ICU transfer and discharge (*P* < 0.05). No statistically significant distinctions were noted in the length of hospital stay or quality of life scores fifteen days post-discharge.

**Conclusion:**

Hyperinsulinemia adversely affects the recovery process of patients with Hypertriglyceridemia-induced acute pancreatitis.

## 1 Background

Acute pancreatitis is one of the most common acute abdomen in clinic. Its main manifestations include abdominal tenderness, dyspepsia, nausea and vomiting, which may cause serious consequences if not treated in time. Studies have shown that the occurrence of acute pancreatitis has a huge negative impact on people’s health and economic ability (1).

A diagnosis of acute pancreatitis is established when a patient exhibits at least two of the following criteria: characteristic abdominal pain, elevated levels (three times or more the upper limit of normal) of pancreatic enzymes (amylase and/or lipase), or imaging results demonstrating specific abnormalities (2). Following a diagnosis, the optimal treatment strategy within the first 72 hours predominantly includes fluid rehydration and enteral nutrition support (3). Approximately 70% to 80% of pancreatitis patients attain symptom control with prompt diagnosis and systematic treatment, resulting in a relatively mild prognosis (4). Hypertriglyceridemic pancreatitis (HP) represents 2% to 4% of all pancreatitis cases (5), indicating a notable increase (6). Hypertriglyceridemic pancreatitis typically follows a more severe disease trajectory, with an increased risk of sustained organ failure. Treatment may necessitate insulin administration to manage the condition effectively (7).

Insulin resistance(IR) is characterized as the phenomenon that at normal plasma insulin levels, target tissues fail to demonstrate the usual synergistic hypoglycemic effects, inhibit endogenous glucose production, suppress lipolysis, and promote glucose uptake. To compensate for insulin resistance, the body may increase its insulin production, resulting in elevated fasting plasma insulin levels, potentially progressing to hyperinsulinemia (8, 9). Hyperinsulinemia, characterized by excessive circulating insulin (10), is positively associated with obesity and serves as a clinical indicator in a subset of patients with insulin resistance (11). Research indicates that hyperinsulinemia is currently a greater contributor to the development of type 2 diabetes than insulin resistance (12). Insufficient insulin function triggers compensatory hyperinsulinemia, necessitating continuous insulin secretion by islet beta cells. This process hastens the deterioration of pancreatic beta cells, potentially inducing apoptosis (13), and impairing pancreatic function. Long-term chronic inflammation induces hyperinsulinemia, which in turn promotes inflammation (14).

Despite the established connection between hyperinsulinemia, fat metabolism, and pancreatic function, research on the influence of hyperinsulinemia on short-term outcomes in patients with Hypertriglyceridemia-induced acute pancreatitis remains insufficient. The objective of this research is to examine the impact of hyperinsulinemia on relevant laboratory indicators and disease severity following standard treatment in patients with acute pancreatitis caused by hyperlipidemia, thereby providing a novel theoretical foundation for managing this medical condition.

## 2 Clinical data and experimental methodologies

### 2.1 Introduction

This study conducted a retrospective analysis of ninety-two patients diagnosed with acute pancreatitis and hyperlipidemia admitted to the surgery department at Wuhu Hospital of Traditional Chinese Medicine, affiliated with Anhui University of Chinese Medicine, from January 2020 to April 2023. Patients were categorized into two groups according to OGTT, glucagon-stimulating C-peptide test and clinical manifestations: the Hyperinsulinemia (HINS) group, with forty-four patients, and the Non-hyperinsulinemia (NHINS) group, with forty-eight patients. Statistical analysis revealed no significant differences in baseline characteristics between the two groups (p > 0.05), indicating their comparability.

### 2.2 Criteria for inclusion and exclusion

The study enrolled 92 patients who received treatment for Hypertriglyceridemia-induced acute pancreatitis at the Department of Surgery, Wuhu Hospital of Traditional Chinese Medicine, affiliated with Anhui University of Chinese Medicine, from January 2020 to April 2023. Acute pancreatitis (AP) was diagnosed based on symptoms of abdominal pain, elevated levels of blood amylase/lipase, and/or typical radiological findings. Hyperlipidemia acute pancreatitis (HLAP) was defined as a triglyceride (TG) level exceeding 5.6mmol/L after excluding other potential causes. Patients under 18 years, pregnant individuals, and those with pancreatic cancer or chronic pancreatitis were excluded from the study. Key clinical parameters such as fasting blood glucose (GLU), total cholesterol (TC), triglycerides (TG), white blood cells (WBC), and high-sensitivity C-reactive protein (CRP) were recorded at specific intervals: one day before lipid-lowering intervention and two days, three to four days, and five to seven days post-intervention.

The diagnostic criteria for acute uncomplicated pancreatitis and mild acute pancreatitis adhere to the standards for acute pancreatitis diagnosis, meeting any of these conditions: no organ failure, no local or systemic complications, and Ranson score < 3 points. These criteria are based on the 2013 Chinese Guidelines for the Diagnosis and Treatment of Pancreatitis. The diagnostic criteria for mild acute pancreatitis are consistent with those for acute pancreatitis, requiring the fulfillment of any of the following: no organ failure, no local or systemic complications, Ranson score < 3 points, Acute Physiology And Chronic Health Evaluation (APACHE) score < 8, Bedside Index For Severity In Acute Pancreatitis (BISAP) < 3 points, or Modified CT Severity Index (MCTSI) score < four points.

Quality of life (QOL) scores were recorded upon admission and within 15 days of discharge using the Moorehead-Ardelt Quality of Life Questionnaire II. The Atlanta Grading standards have categorized the severity of acute pancreatitis into three levels: mild acute pancreatitis (MAP), moderately severe acute pancreatitis (MSAP), and severe acute pancreatitis (SAP). This classification is based on pancreatic necrosis, peripancreatic fluid collections, organ failure, and mortality. Organ failure is characterized by respiratory insufficiency (PaO2/FiO2 ≤300 mmHg), renal failure (serum creatinine ≥170 μmol/L after rehydration), and/or shock (SBP ≤90 mmHg). Additionally, the duration of hospital stay and ICU days are utilized to evaluate outcomes.

The severity of acute pancreatitis was evaluated using the Ranson, BISAP, APACHE-II, and Marshall scoring systems. Ranson scores are applied within the first 24 hours following symptom onset. BISAP scores assess the risk of persistent organ failure in acute pancreatitis patients. An APACHE-II scores of 9 or higher signifies severe pancreatitis. The modified Marshall scoring system assesses pancreatic severity while reducing the impact of renal function on the statistical outcomes (15–20).

### 2.3 Improved gathering of patient information and clinical data, emphasizing ethical considerations

Patient clinical histories, including age, sex, body mass index (BMI), alcohol consumption, diabetes mellitus (DM), and fatty liver status, were retrospectively documented. Patients with a history of heavy alcohol use, defined as consuming ≥40 g/d of alcohol per day for ≥5 years, were excluded from the study due to alcohol-related etiology (21). The levels of serum calcium (Ca), C-reactive protein (CRP), white blood cells (WBC), and triglycerides (TG) were measured.

The initial management of hypertriglyceridemic pancreatitis involves symptomatic measures such as fasting, fluid resuscitation, analgesia, and nutritional support. Additionally, targeted interventions to lower serum triglyceride levels were employed post-initial treatment. These interventions involved insulin infusion, heparin administration, and anti-hyperlipidemic medications. There was no difference in treatment between the two groups. The retrospective observational study protocol adhered to the guidelines outlined in the Declaration of Helsinki.

## 3 Methods of statistical analysis

The data were processed using Graphpad Prism9.5.1 statistical software. Measurement data were presented the mean ± SD, and the t-test was employed for normally distributed data while the rank sum test was used for non-normally distributed data. Count data are presented as percentages and analyzed using the χ2 test. A statistically significant difference was P<0.05 at a significance level of α=0.05.

## 4 Results

### 4.1 Baseline characteristics

**Figure 1:**
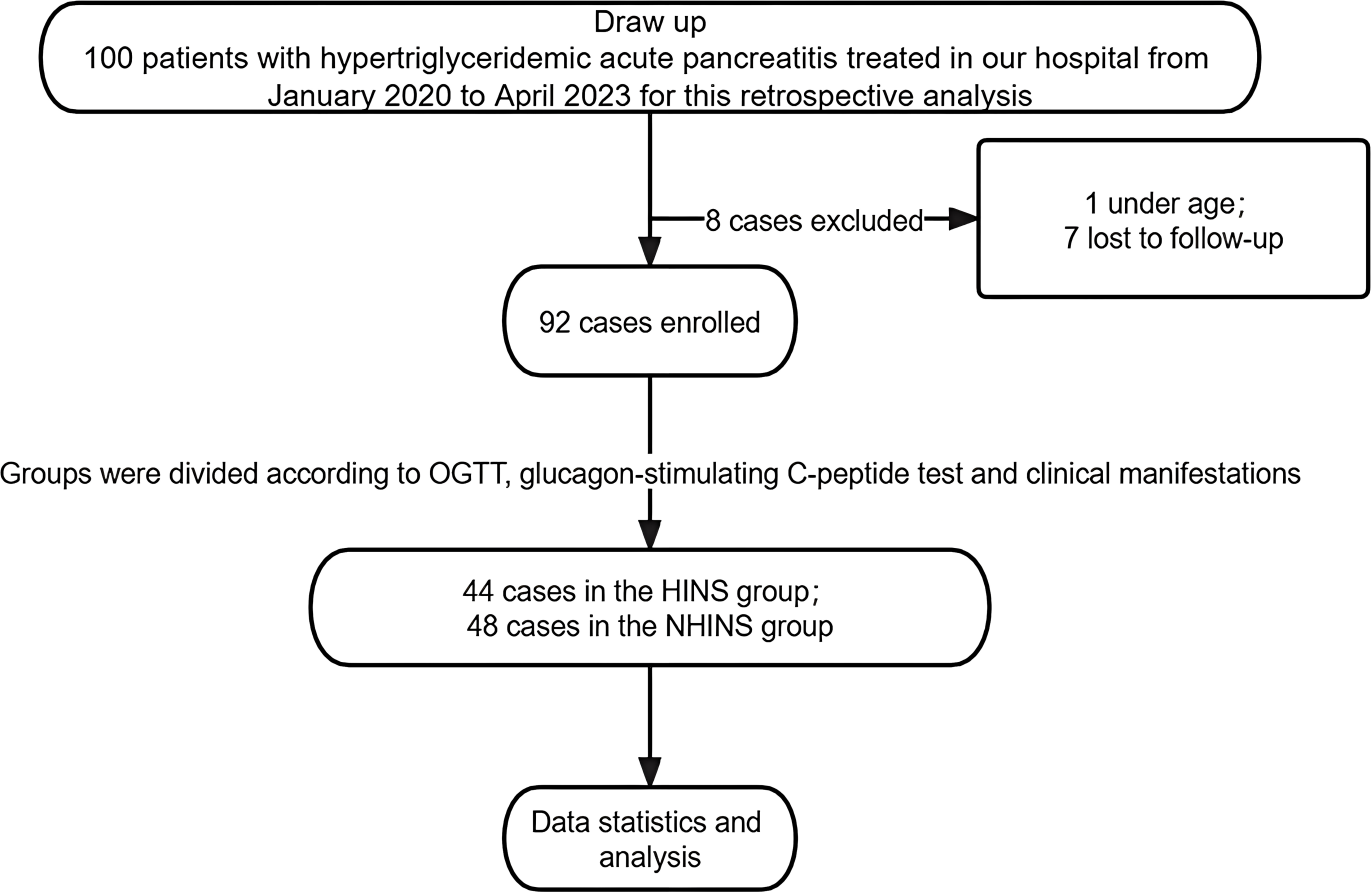
Study flowchart.

The retrospective study comprised 100 patients, with one individual under 18 and seven others with incomplete follow-up excluded from the analysis. These excluded patients were not included in the scope of this research. Patients with FBG > 7mmol/L or OGTT ≥11.1mmol/L, and C-peptide level significantly higher than the normal value, and insulin secretion peak delayed after oral glucose were diagnosed as hyperinsulinemia and included in HINS (n=44), while those without hyperinsulinemia (n=48) were classified as NHINS based on oral glucose tolerance test (OGTT), glucagon-stimulating C-peptide test, and clinical manifestations. **Figure 1** illustrates the detailed methodology employed in this study.

**Table 1** displays the laboratory data and basic information for the two patient groups. The retrospective analysis included age, gender, body mass index (BMI), history of fatty liver disease, quality of life score at admission, blood lipid index, blood glucose index, and inflammatory response index. Statistical comparisons indicated no significant differences between the two groups at baseline, except fasting insulin levels.

**Table 1:** Baseline characteristics.

Variables	HINS (44)	NHINS (48)	P
Men/Women	8/36	6/42	0.5641
Age,y	49.05 ± 6.45	51.15 ± 5.30	0.0904
BMI, kg/m^2^	27.5 ± 3.3	28.0 ± 4.2	0.5297
QOL score	0.23 ± 0.79	0.19 ± 0.98	0.8308
FBG (mmol/L)	9.6 ± 1.7	9.1 ± 1.5	0.1375
Fatty liver, N (%)	38(84.1%)	40(83.3%)	0.7761
Disease severity, N (%)			0.2583
MAP	27(61.4%)	29(60.4%)	
MSAP	13(29.5%)	18(37.5%)	
SAP	4(9.1%)	1(2.0%)	
TG (mmol/L)	10.3 ± 4.2	9.7 ± 3.9	0.4792
TC (mmol/L)	20.7 ± 11.5	17.4 ± 12.5	0.1922
Ca, median (mmol/L)	2.13 ± 0.2	2.12 ± 0.21	0.8160
CRP, mg/L	17.50 ± 4.47	15.68 ± 6.31	0.1163
WBC (10^9)	13.76 ± 4.13)	13.01 ± 5.11	0.4373
HbA1c(%)	6.70 ± 1.22	6.60 ± 1.15	0.7333
FINS (mμ/L)	30.84 ± 13.75	13.96 ± 2.61	P<0.0001
HOMA-IR	9.42 ± 3.51	6.19 ± 2.94	P<0.0001

Data are mean (SD) or %.

BMI, body mass index; FBG, fasting blood glucose; TG, triglyceride; TC, total cholesterol; Ca, calcium; CRP, C-reactive protein; WBC, white blood cell; HbA1c, glycosylated hemoglobin; FINS, fasting insulin.

### 4.2 Prognostic indicators

#### 4.2.1 Lipid Level

During days 5-7, the TG levels showed a significant decline, with the HINS group recording TG levels of 8.1 (3.5) and the NHINS group showing 7.5 (2.3). No statistically significant difference was observed between the two groups during this period. However, notable differences in TG levels were observed between the two groups at baseline and during days 5-7 (**Table 2**).

**Table 2:** Lipid level.

Indicators	HINS (44)	NHINS (48)	P
TG (mmol/L), pre-intervention	10.3 ± 4.2	9.7 ± 3.9	0.4792
TG (mmol/L), D5-D7	8.1 ± 3.5	7.5 ± 2.3	0.3300
P(pre, D5-D7)	0.0091	0.0011	

#### 4.2.2 Blood glucose level

The initial fasting blood glucose (FBG) levels were 9.6 (1.7) mmol/L for the HINS group and 9.1 (1.5) mmol/L for the NHINS group, showing a significant difference from the FBG levels observed on day 2. In addition, the decline of FBG data in the NIHINS group was more pronounced (**Table 3**).

**Table 3:** Blood glucose lever.

Indicators	HINS (44)	NHINS (48)	P
FBG (mmol/L), pre-intervention	9.6 ± 1.7	9.1 ± 1.5	0.1375
FBG (mmol/L), D2	9.0 ± 1.3	7.4 ± 0.8	P<0.0001
FBG (mmol/L), D3-D4	9.1 ± 1.5	7.3 ± 0.9	P<0.0001
FBG (mmol/L), D5-D7	9.3 ± 1.2	7.5 ± 1.1	P<0.0001

#### 4.2.3 The short-term prognosis and rate of recurrence

Hospital stay duration was comparable between the two groups, showing no significant statistical difference. The study documented readmissions within 30 days after discharge for patients with recurrent hypertriglyceridemia-induced acute pancreatitis in both groups. Among the HINS group, 4 patients (9.1%) were readmitted, whereas no readmissions were reported in the NHINS group (0%) (P < 0.05), highlighting a significant difference between the two groups. A total of 7 patients in the HINS group and 1 patient in the NHINS group were transferred to the ICU due to severe conditions, revealing a notable distinction between the two groups (**Table 4**).

**Table 4:** The short-term prognosis and rate of recurrence.

Indicators	HINS (44)	NHINS (48)	P
Hospital days, days	10.18 ± 4.25	9.56 ± 5.17	0.5335
Readmission, N (%)	4(9.1%)	0(0%)	0.0486
ICU need, N (%)	7(15.9%)	1(2.0%)	0.0257

#### 4.2.4 The quality of life

The quality-of-life scores 15 days after discharge were recorded for both groups. Initially, the average quality of life scores was 0.23 (0.79) for the HINS group and 0.19 (0.98) for the NHINS group. Fifteen days after discharge, these scores improved to 1.05 (0.59) for the HINS group and 1.09 (0.68) for the NHINS group, with no significant statistical difference. Importantly, there was a significant improvement in the quality-of-life scores for all patients at the 15-day mark post-discharge compared to their scores before treatment (**Table 5**).

**Table 5:** The quality of life.

Indicators	HINS (44)	NHINS (48)	P
QOL score, pre-intervention	0.23 ± 0.79	0.19 ± 0.98	0.8308
QOL score, post-discharge	1.05 ± 0.59	1.09 ± 0.68	0.7648
P (pre, post)	P<0.0001	P<0.0001	

#### 4.2.5 The comparison of scoring systems

The Ranson, BISAP, APACHE-II, and Marshall scores were documented for both the HINS and NHINS groups upon discharge. Subsequent calculations revealed notable differences in these scores across the two groups (P < 0.005) (**Table 6**).

**Table 6:** Scoring systems.

Indicators	HINS (44)	NHINS (48)	P
Ranson score	4.4 ± 1.8	3.4 ± 0.9	0.001
BISAP score	1.8 ± 0.8	1.3 ± 0.6	0.001
APACHE-II score	5.6 ± 1.1	4.2 ± 0.6	P<0.0001
modified Marshall score	1 ± 0.52	0.75 ± 0.35	0.0077

## 5 Topic under discussion

The primary causes of acute pancreatitis include pancreatic hyperstimulation and obstruction of the pancreaticobiliary duct, leading to increased pancreatic duct pressure and trypsin reflux. Acute pancreatitis arises when the mechanisms to prevent trypsinogen activation or reduce trypsin activity are overwhelmed (22). The predominant factor in AP is a disruption in lipid metabolism pathways. Hyperlipidemia has become the second most common cause of AP, surpassing alcohol, with an increasing prevalence of Hypertriglyceridemia-induced pancreatitis cases. This trend indicates that genes involved in lipid metabolism may affect the risk of secondary infectious pancreatic necrosis in AP patients (23). Multiple factors influence Pancreatitis. Over the past decade, numerous studies have demonstrated that acute pancreatitis can result in metabolic complications (such as exocrine pancreatic dysfunction and new-onset diabetes), regardless of the severity of the initial condition (24).

The management of acute pancreatitis is complex, involving various levels of severity and clinical progression. However, timely and aggressive fluid resuscitation, combined with early initiation of enteral nutrition, has been shown to effectively reduce mortality rates and the occurrence of infections (25). Studies have shown that the progression and severity of remission in acute pancreatitis is driven by local and systemic inflammation (26). Hypertriglyceridemic pancreatitis, a subtype of acute pancreatitis, is initiated by the enzymatic activity of pancreatic lipase on excess fat, producing fatty acids. The subsequent accumulation of lipid toxicity induces robust inflammatory responses (27). IIn addition, hypertriglyceridemia will increase the production of intracellular reactive oxygen species, accelerate the production of inflammatory factors, recruit and activate more inflammatory cells, and further aggravate the inflammatory response (28, 29).

The mortality rate and incidence of organ necrosis in acute pancreatitis are positively correlated with the severity of hyperlipidemia (30). Consequently, triglyceride levels serve as a critical supplementary parameter in the clinical management of acute pancreatitis. Regular monitoring of triglyceride and serum glucose levels is essential during insulin therapy, and discontinuation of intravenous insulin is advised once serum triglyceride levels decrease to below 500mg/dL. The secretion of insulin by pancreatic beta cells plays a pivotal role in regulating glucose, amino acid, and free fatty acid metabolism in the human body (31–33). Maintaining an optimal insulin concentration homeostasis relies on a negative feedback regulatory system. Disruption of this mechanism can result in inadequate or excessive insulin secretion symptoms.

Insulin resistance and the resulting hyperinsulinemia are major manifestations and pathophysiological factors of metabolic syndrome (34, 35). This relationship between insulin resistance and obesity further exacerbates the condition. Both obesity and insulin resistance play a significant role in triggering and worsening disturbances in lipid metabolism, particularly hyperlipidemia (36). Therefore, improving both obesity and insulin resistance is considered one of the treatment approaches for acute pancreatitis with high triglyceride levels. Managing hyperinsulinemia involves both pharmacological and non-pharmacological interventions (37). Pharmacological options for diabetes treatment include sulfonylureas, glucagon-like peptide 1 (GLP-1) agonists, and dipeptidyl peptide-4 (DPP-4) inhibitors (38); while non-pharmacological strategies involve regulating dietary energy intake, increasing physical activity, and enhancing energy expenditure (39). Although innovative and effective, non-drug treatments require a strong sense of self-discipline and regularity (40, 41), making them unsuitable for acute stages of pancreatitis.

However, this study has limitations, including a small sample size leading to biased sample selection, lack of long-term follow-up on the effects of pancreatitis treatment, and the inability to adjust the study based on varying serum triglyceride concentrations. Therefore, future research should focus on these aspects for further exploration.

## 6 Conclusion

In summary, symptomatic treatment was administered to both groups of patients with hyperlipidemia pancreatitis after hospitalization, leading to significant improvements in clinical symptoms and laboratory indicators. However, due to impaired pancreatic function, patients in the HINS group experienced slower recovery and disease aggravation, negatively impacting their condition. Following conventional treatment, patients in the NHINS group showed milder abdominal signs and blood test results than those in the HINS group. Hospital stay duration and quality of life after discharge were not significantly affected in the HINS group.

## Data Availability

The raw data supporting the conclusions of this article will be made available by the authors, without undue reservation.

## References

[1] Zhu S Ding Z. Acute pancreatitis and metabolic syndrome: genetic correlations and causal associations. Endocrine. (2024) 2:380–7. doi: 10.1007/s12020-023-03584-4, PMID: 37922090

[2] Zheng CB Zheng ZH Zheng YP. Therapeutic plasma exchange for hyperlipidemic pancreatitis: Current evidence and unmet needs. World J Clin cases. (2021) 9:5794–803. doi: 10.12998/wjcc.v9.i21.5794, PMID: 34368298 PMC8316951

[3] Lee PJ Papachristou GI. New insights into acute pancreatitis. Nat Rev Gastroenterol Hepatol. (2019) 16:479–96. doi: 10.1038/s41575-019-0158-2, PMID: 31138897

[4] Gardner TB. Acute pancreatitis. Ann Intern Med. (2021) 174(2):ITC17–32. doi: 10.7326/AITC202102160, PMID: 33556276

[5] James TW Crockett SD. Management of acute pancreatitis in the first 72 hours. Curr Opin Gastroenterol. (2018) 34:330–5. doi: 10.1097/MOG.0000000000000456, PMID: 29957661 PMC6245573

[6] Bollen TL. Acute pancreatitis: international classification and nomenclature. Clin Radiol. (2016) 71:121–33. doi: 10.1016/j.crad.2015.09.013, PMID: 26602933

[7] Yang AL McNabb-Baltar J. Hypertriglyceridemia and acute pancreatitis. Pancreatology. (2020) 20:795–800. doi: 10.1016/j.pan.2020.06.005, PMID: 32571534

[8] Petersen MC Shulman GI. Mechanisms of insulin action and insulin resistance. Physiol Rev. (2018) 98:2133–223. doi: 10.1152/physrev.00063.2017, PMID: 30067154 PMC6170977

[9] da Silva AA do Carmo JM Li X Wang Z Mouton AJ Hall JE. Role of hyper-insulinemia and insulin resistance in hypertension: metabolic syndrome revisited. Can J Cardiol. (2020) 36:671–82. doi: 10.1016/j.cjca.2020.02.066, PMID: 32389340 PMC7219403

[10] Templeman NM Skovsø S Page MM Lim GE Johnson JD. A causal role for hyperinsulinemia in obesity. J Endocrinol. (2017) 232:R173–83. doi: 10.1530/JOE-16-0449, PMID: 28052999

[11] Yue Z Qian L Jin Y Xia Y Sha H Wu Q. Hyperinsulinemia influences the short-term efficiency of laparoscopic sleeve gastrectomy for patients with obesity and insulin resistance. Diabetes Metab Syndr Obes. (2023) 16:1745–53. doi: 10.2147/DMSO.S411440, PMID: 37334184 PMC10276567

[12] Erion KA Corkey BE. Hyperinsulinemia:a cause of obesity? Curr Obes Rep. (2017) 6:178–86. doi: 10.1007/s13679-017-0261-z, PMID: 28466412 PMC5487935

[13] Mezza T Cinti F Cefalo CMA Pontecorvi A Kulkarni RN Giaccari A. β-cell fate in human insulin resistance and type 2 diabetes:A perspective on islet plasticity. Diabetes. (2019) 68:1121–9. doi: 10.2337/db18-0856, PMID: 31109941 PMC6905483

[14] Zhang AMY Wellberg EA Kopp JL Johnson JD. Hyperinsulinemia in obesity, inflammation, and cancer. Diabetes Metab J. (2021) 45:285–311. doi: 10.4093/dmj.2020.0250, PMID: 33775061 PMC8164941

[15] Banks PA Bollen TL Dervenis C Gooszen HG Johnson CD Sarr MG. Classification of acute pancreatitis–2012:revision of the Atlanta classification and definitions by international consensus. Gut. (2013) 62:102–11. doi: 10.1136/gutjnl-2012-302779, PMID: 23100216

[16] Abu Omar Y Attar BM Agrawal R Randhawa T Majeed M Wang Y. Revised marshall score: A new approach to stratifying the severity of acute pancreatitis. Dig Dis Sci. (2019) 64:3610–5. doi: 10.1007/s10620-019-05719-y, PMID: 31286346

[17] Gliem N Ammer-Herrmenau C Ellenrieder V Neesse A. Management of severe acute pancreatitis: an update. Digestion. (2021) 102:503–7. doi: 10.1159/000506830, PMID: 32422634 PMC8315686

[18] Karabuga B Gemcioglu E Konca Karabuga E Baser S Ersoy O. Comparison of the predictive values of CRP, CRP/albumin, RDW, neutrophil/lymphocyte, and platelet/lymphocyte levels in determining the severity of acute pancreatitis in patients with acute pancreatitis according to the BISAP score. Bratisl Lek Listy. (2022) 123:129–35. doi: 10.4149/BLL_2022_020, PMID: 35065589

[19] Woo SM Noh MH Kim BG Hsing CT Han JS Ryu SH. Comparison of serum procalcitonin with Ranson, APACHE-II, Glasgow and Balthazar CT severity index scores in predicting severity of acute pancreatitis. Korean J Gastroenterol. (2011) 58:31–7. doi: 10.4166/kjg.2011.58.1.31, PMID: 21778801

[20] Harshit Kumar A Singh Griwan M. A comparison of APACHE II, BISAP, Ranson’s score and modified CTSI in predicting the severity of acute pancreatitis based on the 2012 revised Atlanta Classification. Gastroenterol Rep (Oxf). (2018) 6:127–31. doi: 10.1093/gastro/gox029, PMID: 29780601 PMC5952961

[21] Dong X Pan S Zhang D Hong W Chen T Zhang B. Hyperlipemia pancreatitis onset time affects the association between elevated serum triglyceride levels and disease severity. Lipids Health Dis. (2022) 21:49. doi: 10.1186/s12944-022-01656-4, PMID: 35637538 PMC9153118

[22] Wang GJ Gao CF Wei D Wang C Ding SQ. Acute pancreatitis:etiology and common pathogenesis. World J Gastroenterol. (2009) 15:1427–30. doi: 10.3748/wjg.15.1427, PMID: 19322914 PMC2665136

[23] Wolbrink DRJ Kolwijck E Ten Oever J Horvath KD Bouwense SAW Schouten JA. Management of of infected pancreatic necrosis in the intensive care unit:a narrative review. Clin Microbiol Infect. (2020) 26:18–25. doi: 10.1016/j.cmi.2019.06.017, PMID: 31238118

[24] Petrov MS. Metabolic trifecta after pancreatitis: exocrine pancreatic dysfunction, altered gut microbiota, and new-onset diabetes. Clin Transl Gastroenterol. (2019) 10:e00086. doi: 10.14309/ctg.0000000000000086, PMID: 31609744 PMC6884355

[25] Mederos MA Reber HA Girgis MD. Acute pancreatitis: A review. JAMA. (2021) 325:382–90. doi: 10.1001/jama.2020.20317, PMID: 33496779

[26] Hansen SEJ Madsen CM Varbo A Nordestgaard BG. Low-grade inflammation in the association between mild-to-moderate hypertriglyceridemia and risk of acute pancreatitis: A study of more than 115000 individuals from the general population. Clin Chem. (2019) 2:321–32. doi: 10.1373/clinchem.2018.294926, PMID: 30518661

[27] Saluja A Dudeja V Dawra R Sah RP. Early intra-acinar events in pathogenesis of pancreatitis. Gastroenterology. (2019) 156:1979–93. doi: 10.1053/j.gastro.2019.01.268, PMID: 30776339

[28] Qiu M Zhou X Zippi M Goyal H Basharat Z Jagielski M. Comprehensive review on the pathogenesis of hypertriglyceridaemia-associated acute pancreatitis. Ann Med. (2023) 2:2265939. doi: 10.1080/07853890.2023.2265939, PMID: 37813108 PMC10563627

[29] Ávila-Escalante ML Coop-Gamas F Cervantes-Rodríguez M Méndez-Iturbide D Aranda-González II. The effect of diet on oxidative stress and metabolic diseases-Clinically controlled trials. J Food Biochem. (2020) 5:e13191. doi: 10.1111/jfbc.13191, PMID: 32160647

[30] Zhang R Deng L Jin T Zhu P Shi N Jiang K. Hypertriglyceridaemia-associated acute pancreatitis:diagnosis and impact on severity. HPB (Oxford). (2019) 21:1240–9. doi: 10.1016/j.hpb.2019.01.015, PMID: 30885545

[31] Norton L Shannon C Gastaldelli A DeFronzo RA. Insulin:The master regulator of glucose metabolism. Metabolism. (2022) 129:155142. doi: 10.1016/j.metabol.2022.155142, PMID: 35066003

[32] Liu R Zhang L You H. Insulin resistance and impaired branched-chain amino acid metabolism in Alzheimer’s disease. J Alzheimers Dis. (2023) 93:847–62. doi: 10.3233/JAD-221147, PMID: 37125547

[33] Stefanovski D Punjabi NM Boston RC Watanabe RM. Insulin action, glucose homeostasis and free fatty acid metabolism : insights from a novel model. Front Endocrinol (Lausanne). (2021) 12:625701. doi: 10.3389/fendo.2021.625701, PMID: 33815283 PMC8010655

[34] Fahed G Aoun L Bou Zerdan M Allam S Bou Zerdan M Bouferraa Y. Metabolic syndrome: updates on pathophysiology and management in 2021. Int J Mol Sci. (1970) 23(2):786. doi: 10.3390/ijms23020786, PMID: 35054972 PMC8775991

[35] Gluvic Z Zaric B Resanovic I Obradovic M Mitrovic A Radak D. Link between metabolic syndrome and insulin resistance. Curr Vasc Pharmacol. (2017) 1:30–9. doi: 10.2174/1570161114666161007164510, PMID: 27748199

[36] Vergès B Vantyghem MC Reznik Y Duvillard L Rouland A Capel E. Hypertriglyceridemia results from an impaired catabolism of triglyceride-rich lipoproteins in PLIN1-related lipodystrophy. Arterioscler Thromb Vasc Biol. (2024) 8:1873–83. doi: 10.1161/ATVBAHA.124.320774, PMID: 38899472

[37] Ferguson D Finck BN. Emerging therapeutic approaches for the treatment of NAFLD and type 2 diabetes mellitus. Nat Rev Endocrinol. (2021) 17:484–95. doi: 10.1038/s41574-021-00507-z, PMID: 34131333 PMC8570106

[38] Padhi S Nayak AK Behera A. Type II diabetes mellitus:a review on recent drug based therapeutics. BioMed Pharmacother. (2020) 131:110708. doi: 10.1016/j.biopha.2020.110708, PMID: 32927252

[39] Mirabelli M Chiefari E Arcidiacono B Corigliano DM Brunetti FS Maggisano V. Mediterranean diet nutrients to turn the tide against insulin resistance and related diseases. Nutrients. (2020) 12:1066. doi: 10.3390/nu12041066, PMID: 32290535 PMC7230471

[40] Banaszak M Górna I Przysławski J. Non-pharmacological treatments for insulin resistance : effective intervention of plant-based diets-A critical review. Nutrients. (2022) 14:1400. doi: 10.3390/nu14071400, PMID: 35406013 PMC9002735

[41] Napoleão A Fernandes L Miranda C Marum AP. Effects of calorie restriction on health span and insulin resistance:Classic calorie restriction diet vs. Ketosis-inducing diet. Nutrients. (2021) 13:1302. doi: 10.3390/nu13041302, PMID: 33920973 PMC8071299

